# Implicit expression of uncertainty – suggestion of an empirically derived framework

**DOI:** 10.1186/s12909-020-1990-3

**Published:** 2020-03-20

**Authors:** Julia Gärtner, Pascal O. Berberat, Martina Kadmon, Sigrid Harendza

**Affiliations:** 1 III. Department of Internal Medicine, University Medical Center Hamburg-Eppendorf, Hamburg, Germany; 2 TUM Medical Education Center, School of Medicine, Technical University of Munich, Munich, Germany; 3 Faculty of Medicine, University of Augsburg, Augsburg, Germany

**Keywords:** Certainty, Clinical reasoning, Communication, Competence-based assessment, Handoff, Uncertainty

## Abstract

**Background:**

Uncertainty occurs in physicians’ daily work in almost every clinical context and is also present in the clinical reasoning process. The way physicians communicate uncertainty in their thinking process during handoffs is crucial for patient safety because uncertainty has diverse effects on individuals involved in patient care. Dealing with uncertainty and expressing uncertainty are important processes in the development of professional identity of undergraduate medical students. Many studies focused on how to deal with uncertainty and whether uncertainty is explicitly expressed. Hardly any research has been done regarding implicit expression of uncertainty. Therefore, we studied the ways in which medical students in the role of beginning residents implicitly express uncertainty during simulated handoffs.

**Methods:**

Sixty-seven advanced undergraduate medical students participated in a simulated first day of residency including a consultation hour, a patient management phase with interprofessional interaction, and a patient handoff. We transcribed the videographed handoffs verbatim and extracted language with respect to expression of uncertainty using a grounded theory approach. Text sequences expressing patient related information were analyzed and coded with respect to language aspects which implicitly modified plain information with respect to increasing or decreasing uncertainty. Concepts and categories were developed and discussed until saturation of all aspects was reached.

**Results:**

We discovered a framework of implicit expressions of uncertainty regarding diagnostic and treatment-related decisions within four categories: “Statement”, “Assessment”, “Consideration”, and “Implication”. Each category was related to either the subcategory “Actions” or “Results” within the diagnostic or therapeutic decisions. Within each category and subcategory, we found a subset of expressions, which implicitly attenuated or strengthened plain information thereby increasing uncertainty or certainty, respectively. Language that implicitly attenuated plain information belonged to the categories questionable, incomplete, alterable, and unreliable while we could ascribe implicit strengtheners to the categories assertive, adequate, focused, and reliable.

**Conclusions:**

Our suggested framework of implicit expression of uncertainty may help to raise the awareness for expression of uncertainty in the clinical reasoning process and provide support for making uncertainty explicit in the teaching process. This may lead to more transparent communication processes among health care professionals and eventually to improved patient safety.

## Background

The focused, oral case presentation is one of the key elements of clinical reasoning in patient care [1]. Different models have been proposed to teach and assess focused case presentation [2–5]. At the same time, uncertainty is a constant companion in medical practice. In different health care systems and under different cultural circumstances there may be differences in expressing uncertainty or certainty [6]. Uncertainty increases stress and can potentially hamper medical decision-making [7], leads to ordering more tests [8], and causes unnecessary admissions and even harm patients [9]. Therefore, uncertainty needs to be addressed and explicitly expressed during undergraduate and postgraduate medical education, especially in focused case presentations [10]. Uncertainty has been defined as the subjective perception of a physician’s inability to provide an accurate explanation of a patient’s health problem [11]. Acknowledging and expressing the “certainty of uncertainty” in medicine could improve patient care and would offer a complementary concept to current medical education principles which focus on verbally expressing knowledge and certainty [12]. In an environment where medical students and junior physicians constantly have to prove their knowledge and where good grades are given for correct answers, acknowledgment of uncertainty is often suppressed or hidden by learners [13]. Lingard et al. found that medical students even developed strategies to mask their uncertainty [14], which could even have harmful consequences when such behaviour occurs in real physician-patient situations. If, on the other hand, students learn to openly express their uncertainty, e.g. by using the six-steps SNAPPS technique for case presentation (S: summarize history and findings, N: narrow the differential, P: probe preceptors about uncertainties, P: plan management, S: select case-related issues for self-study), the whole teaching process between supervisor and trainee may improve [15].

Moreover, in a medical culture, where medical students and physicians feel vulnerable when expressing uncertainty and therefore internalize and mask it [16], perhaps because it can lead to embarrassment or patient dissatisfaction [17], there is a strong need to acknowledge uncertainty and embrace its presence for the patients’ safety. In the United Kingdom and a few other countries medical uncertainty is already regarded as one of the core clinical competencies for medical graduates and trainees [18–21]. A shift in culture could be facilitated by using a different way of speaking, e.g. by stating “hypotheses” rather than “diagnoses” [22]. Medical students should exercise as early as possible in using language and reasoning skills to recognize, evaluate, and mitigate uncertainty when they encounter it [23].

Before such a concept of revealing and appraising uncertainty can be applied in teaching, it is important to gain an insight into uncertainty when it is not explicitly expressed by the student. A critical moment in such a context of potential uncertainty and patient safety are accurate case presentations during the handoff between shifts [24]. Therefore, the aim of our study was to qualitatively explore language used by advanced undergraduate medical students to implicitly express uncertainty, when handing patients off in the role of a junior resident during a simulated first day of residency.

## Methods

### Study design and participants

Sixty-seven medical students attending year 5 (*n* = 26) and 6 (*n* = 41) of a 6-year undergraduate medical program at three German medical faculties (Hamburg, Oldenburg, and Technical University of Munich) participated in the role of a beginning in a 360-degree competence-based assessment simulating the first day of residency [25]. Participation was voluntary and registration for participation occurred on a first come, first serve basis. The assessment included a consultation hour with five simulated patients, followed by a patient management phase where tests could be ordered and interprofessional interactions took place, and ended with the handoff of the five patients to a real resident. This validated simulation, a formative procedure, provided a near-authentic opportunity for the students to act as physicians with full responsibilities [26].

### Materials and procedure

All handoffs were videotaped and transcribed verbatim. In order to study implicit verbal expression of uncertainty we used a qualitative, explorative research approach based on the concept of grounded theory [27]. A person not involved in this study compiled the transcribed handoffs for analysis to ensure a balanced mix of transcripts with respect to the students’ gender and year in medical school (5th or 6th) to ensure that these criteria would not affect the findings. The coders were blinded to the students’ sociodemographic data of the transcripts which were given to them for analysis.

### Data analysis

We started analysing transcripts from Hamburg Medical Faculty, which provides a vertically-integrated model curriculum for undergraduate medical education. By using MAXQDA 2018 (Release 18.2.0, VERBI GmbH), a software program for qualitative text and multimedia analysis, per text sequences as units of meanings, JG (sociologist) and SH (internist, medical educationalist) individually analysed the language used by the students with reference to implicit expressions of uncertainty or certainty. The individually identified concepts and categories were discussed with POB (surgeon, medical educationalist) and MK (surgeon, medical educationalist). We focused on sequences during the case presentations where students expressed diagnostic and treatment-related decisions they had made for the different patients as well as conclusions they had drawn from test results or other information during the management phase. We excluded information arising from discussions with the residents who were informed about the correct workup for the different patients. We also excluded sequences with explicit expression of uncertainty, e.g. “[ …] with [the decisions for] this [patient] I was unsure.”, “Monitoring [this patient] would certainly not be wrong”. When a student, for instance, expressed to “not know” something we interpreted this as an implicit way of expressing uncertainty by referencing to knowledge. The coders’ professional backgrounds allowed for different perspectives, which expressions appeared to be more or less uncertain or certain. The physicians’ interpretations provided their specific medical perspective and the sociologist’s interpretations focused on general interaction and communication patterns. The first round of coding focused on the effect language had on the student’s expression of plain information. In further rounds, we identified relationships between the characteristics of the codes, reviewed and constantly refined them and thereby specified and sharpened the concepts. By discussing the different perspectives the coders approached agreement of the concepts and categories.

During data analysis the hypothesis arose that the students’ language could vary according to the respective teaching strategies used in the undergraduate curricula of the different medical schools (vertically-integrated versus not vertically-integrated curriculum). Therefore, after the analysis of eight transcripts from the University of Hamburg, when no further codes or categories appeared, we repeat the process with transcripts of the two other medical school, the Medical Faculty of the Technical University Munich (not vertically-integrated curriculum) and the Medical Faculty of the University of Oldenburg (vertically-integrated curriculum in cooperation with the University of Groningen, the Netherlands). The transcripts were again provided in a blinded fashion with respect to a balanced mix of students’ gender and year of medical school. Data collection was stopped after analysing five transcripts from medical students of the Technical University of Munich and two transcripts from medical students of the University of Oldenburg when the data seemed to have reached an acceptable level of saturation for the different categories. With respect to the language-culture effect [28], we translated quotations illustrating the respective categories from German to English keeping the original content and expression including fine nuances intact. The translations were discussed with a native English speaking physician and medical educator not involved in this study. The study was performed in accordance with the Declaration of Helsinki and the Ethics Committee of the Chamber of Physicians (Ethik-Kommission, Ärztekammer Hamburg), Hamburg, confirmed the innocuousness of the study including written consent of participants and anonymized and voluntary participation (reference number: PV3649).

## Results

We discovered a multilayered structure of expressions concerning diagnostic and treatment related decisions resulting in four main categories: “Statement”, “Assessment”, “Consideration”, and “Implication” (Table 1). In each category, we found reports related to either the diagnostic or therapeutic subcategories action or result. For each category and subcategory, we provide examples of plain (unmodified) information. Secondly, examples with language that attenuates information and thereby implicitly increases the degree of uncertainty are displayed. Thirdly, samples of language that strengthens information and thereby implicitly increases the degree of its certainty are included. Exemplary quotes for all categories are shown in Table 1.

**Table 1:**
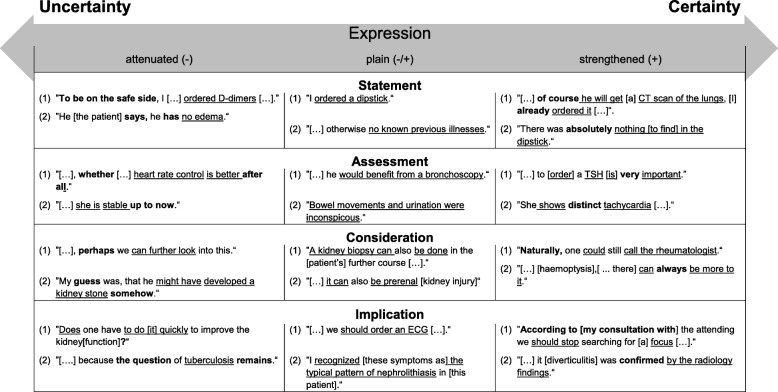
Categories of expressions within the uncertainty-certainty continuum

(1) action related expression, (2) result related expression; underlined: category of expression; bold: implicit expression of uncertainty or certainty

Regarding the four main categories, “Statements” comprised information about the actions a participant had undertaken or refrained from (e.g. I have done/I did not) and about medical results which had been gathered (e.g. it was/they were not). They included the duration or explicit localization of symptoms and the details of laboratory results, expressed with medical terms or explicit values. “Assessments” expressed the appraisal of the characteristics of diagnostic actions and medical findings (e.g. in/conspicuous; un/important; positive/negative; not/good), their dimension (e.g. massive; moderate; light) or how well they fit within a differential diagnosis (e.g. not/matching). “Considerations” included the suggestion of options for further diagnostic or therapeutic actions or their results (e.g. I/we/you can/could). Lastly, “Implications” transferred the meaning of actions or results into possible outcomes (I/we/you; must/have/need/want/would/should; I think/thought; my suspicion/diagnose/idea). We refrained from denominating the use of subjunctive implicit uncertainty, since subjunctive can also function as a form of politeness in a professional context e.g. “one could think about consulting a dermatologist”. Table 1 provides examples representing the diversity of language, which implicitly attenuated or strengthened plain “Statements”, “Assessments”, “Considerations”, and “Implications”, thereby shifting the meaning towards uncertainty or certainty, respectively. Tables 2 and 3 show the different categories of attenuators and strengtheners which we developed from the handoffs.

**Table 2:** Categories of language that implicitily attenuates plain information

Main category	Subcategory + Language	Example
A) Questionable	a) Questions (direct/indirect) • Would you […]? • It probably could [be] […], [couldn’t it?]	“Does one have to do this acutely to improve the kidney function?”
	b) Doubtful • whether • debatable • if it [really] was like this	“It is debatable whether one has to check her for colon cancer.”
	c) Hypothetical • guess • suppose • probably/presumably • perhaps/maybe	“Therefore, it’s maybe an […] abscess.”
B) Incomplete	a) Inconclusive • does not make sense • at a loss/clueless • just did something • hard to say	“I am a bit clueless what the [diagnosis] will be?”
	b) Ambiguous • unclear • somewhat	“The ultrasound [result] remains unclear.”
	c) Unperceived • hard to see/recognize • not very visible	“It’s hard for me to recognize p-waves [in this ECG].”
	d) Absent (finding/experience/knowledge) • outstanding/pending test result • not yet • do not know	“I don’t know whether one has to give her [the aciclovir] i.v.”
C) Alterable	a) Directly modifying • relatively	“She [the patient] is relatively stable. “
	b) Indirectly modifying • at the moment/this minute • at a first glance • currently • right now • quote unquote • thus far/for now • almost • initially	“At the moment [the patient] is stable so far.”
D) Unreliable	a) Expert outside [specific medical] field • according to […] • he/she/it said • he/she possibly has	“She takes two drugs against diabetes which she probably acquired because of steroids, she said.”
	b) Lacking evidence • […] wasn’t in the chart/results	“It was not in the results [of the physical examination].”

**Table 3:** Categories of language that implicitily strengthens plain information

Main category	Subcategory + Language	Example
A) Assertive	a) Instruction (direct/indirect) • look at this • you still need to do	“You would have to order liver enzymes.”
	b) Independent • did something independently • did not discuss something [with the attending] beforehand	“I independently ordered a HR-CT.”
	c) Inevitable • in any case • anyway	“Keep [the patient in hospital] in any case, I would say.”
B) Adequate	a) Coherent • logically/naturally/of course • in principle • straight from the textbook	“Naturally, the rheumatoid factor was elevated.”
	b) Unambiguous • clear/clearly • really • definitely • distinctly	“CRP was 123, hence, a definite sign of infection”.
	c) Perceptible • recognizable • have seen • visible	“[Free peritoneal air] was very visible for me on the X-ray.”
C) Focused	a) Absolute • never • nothing at all • quite • always	“There was absolutely nothing in the dip stick.”
	b) Simple • simply • just like that	“He can simply have a systemic rheumatoid disease.”
	c) Prioritized • first of all / at first • rather • most likely • at least • very important • primarily • the same day • already	“First of all, here are the cANCAs we ordered.”
D) Reliable	a) Medical expert • according to […] • after consultation with […]	“According to [my] consultation with the supervisor we should stop searching for a source of infection.”
	b) Non-medical but insistent expert • […] said several times	“The patient said several time that she tends to fall.”
	c) Evidenced • […] confirmed • […] refuted	“Diverticulitis was confirmed by the radiology result.”

Attenuators (Table 2), which implicitly shifted the meaning of plain information towards uncertainty, made it either “Questionable” (main category A), “Incomplete” (main category B), “Alterable” (main category C), or “Unreliable” (main category D). In the “Questionable” main category A we identified direct or indirect “Questions”, the expression of “Doubt”, and the expression of information as being “Hypothetical” as subcategories. “Incomplete” information (main category B) was expressed as being “Inconclusive”, “Ambiguous”, “Not clearly seen”, or “Absent”. For the “Alterable” main category C expressions were found that directly (“Directly modifying”) or indirectly (“Indirectly modifying”) changed information and thereby limited its validity. In the main category D information became “Unreliable” when reference to an “Expert outside [specific medical] field” was made, e.g. by referring to the patient who is the ‘expert’ of his/her symptoms or by citing a specialist from a medical field other than the disease hypothesized by the student, or to a person from another health profession. Alternatively, the unreliability of Category D was expressed by “Lacking evidence” to prove information. Specific examples for all subcategories are provided in Table 2.

In Table 3, the main categories and subcategories for strengtheners that implicitly shift the meaning of plain information towards certainty are described. Strengtheners include “Assertive” language (main category A), which can be expressed as direct" or indirect “Instruction”. In this category, decisions were made in an “Independent” or “Inevitable” way. In main category B, “Adequate”, information was expressed in a “Coherent”, “Unambiguous”, or “Perceptible” way. “Focused” information (main category C) strengthened the reports either by reducing complexity and making them “Absolute”, “Simple” or by giving “Priority” to certain information. Lastly, “Reliable” sources (main category D) strengthened information by citing opinions of a “Medical expert” or by a “Non-medical but insistent expert”. Information was also strengthened with respect to reliability when it was “Evidenced” in either a confirming or refuting way.

We also identified expressions modified by more than one attenuator or strengthener, e.g.: “[ …] I guessed that it could maybe be herpes.” (combination of two attenuators from the subcategory Ac), “Of course I have already ordered a CT-scan [myself].” (combination of strengtheners Ab and Ac). Additionally, some combinations of an attenuator and a strengthener were detected, which caused ambivalences in the expression or a shift towards the aspect of uncertainty, e.g.: “There is definitely something, but one cannot say, what it exactly is.” (combination of strengthener Bb and attenuator Db), “Of course one could maybe order [liver function tests].” (combination of strengthener Ac and attenuator Ac). Furthermore, we identified the use of the word “actually” as being content dependent and therefore varied with respect to its tendency towards uncertainty or certainty. No additional concepts and categories emerged when transcripts from the medical faculties of the Technical University of Munich and of the University of Oldenburg were added to the analysis.

## Discussion

With the quest for certainty being central to the human psychology [16] it seems to be a natural and professional desire that physicians wish to be as certain as possible when diagnosing or treating patients. At the same time, uncertainty about the progression of a patient’s condition, the best diagnostic steps or the benefit of a particular treatment is are familiar challenges in physicians’ daily work. The tolerance of ambiguity has been shown to be higher in intern and resident physicians than in undergraduate medial students [29] suggesting a professional development in accepting and dealing with the fact that physicians are continuously faced with uncertainty. However, the actual relationship between physicians’ self-assessed certainty and their diagnostic accuracy varies and is context dependent, e.g. when clinical data are inconsistent [30]. Since continuous patient care requires the handoff of information in many varied clinical situations, it seems to be particularly important for the patients’ safety that the physician who receives a handoff report correctly perceives the extent to which the reporting physician feels certain or uncertain about the reported information. In a medical culture where uncertainty is considered to be a weakness [13], learning about the importance of expressing uncertainty in their clinical reasoning process and how to do so [31] remains challenging for medical students. Despite an increasing amount of clinical information and knowledge, clinical decisions remain complex and doubt is present most of the time. Therefore, assessment of clinical reasoning in the context of uncertainty has been proposed as a concept for learning and dealing with clinical reasoning [32].

To date, expression of uncertainty has been studied in case presentations with a focus on its explicit expression providing information about physicians’ patient management and portrayal of uncertainty [14, 15]. In our study, we focused on implicit expression of uncertainty because its detection might be much subtler and could lead to difficulties in patient care if it went unnoticed. Medical students in our study reported about simulated patients’ diagnostic and treatment decisions during simulated handoffs. Their expressions fit into four categories: “Statements” which included facts, or “Assessment”, “Consideration”, and “Implication”, which expressed students’ conclusions and recommendations. Within these four categories relevant to clinical reasoning, we identified a framework consisting of plain information as well as language which attenuated or strengthened information, thereby shifting its content more towards uncertainty or certainty. It is an intriguing finding that “Statements” and “Assessments” extracted from the handoffs implicitly expressed certainty or uncertainty. Interestingly, “Considerations” and “Implications”, which contain largely non-factual information, were stated with enhanced certainty when combined with language that implicitly strengthens the plain information. Implicit expression of uncertainty might have evolved during medical students’ and physicians’ socialization processes. Medical students have reported their fear of seeming uncertain and showed tendencies to deflect criticism and demonstrate competence in case presentations, no matter how confident they actually are [33]. At the same time, parents of pediatric patients rated physicians’ competence in pediatric case vignettes higher and trusted them more when the physicians implicitly communicated uncertainty [34].

While it may be useful in physician-patient communication to express uncertainty in an implicit way, explicit communication of uncertainty between medical professionals in clinical settings seems to be the communication style of choice to reduce medical errors [35]. In medical settings, the perception of implicit enhancement or reduction of certainty is highly variable. For example, a study on phrases expressing diagnostic certainty used in radiology reports showed that there was a wide variety in the use of such phrases, with good agreement among radiologists about the extent of certainty that a particular phrase expressed [36]. In contrast, among radiologists and nonradiologists, only poor concordance was detected with respect to the certainty associated with phrases commonly used in radiology reports [37]. In this study, the greatest variety of interpretation was detected for terms like “probably” (which we identified here as attenuators of plain information in the subcategory “hypothetical” of the main category “questionable”). In the same study the highest agreement was found for terms like “always” or “never” (which occur in our framework as strengtheners of plain information in the subcategory “absolute” of the main category “focused”). Haber and Lingard have shown that medical students learn to present patient cases in a trial and error fashion rather than through teaching of an explicit rhetorical model [38]. This implies that aspects of implicit and explicit expression of certainty and uncertainty and their possible effects on patient treatment and outcome are probably also not taught. Our findings suggest that awareness for the use of language and its implicit expression of uncertainty could be taught to medical students and physicians with the support of our newly described framework.

This framework offers a constructive teaching approach to acknowledge the “certainty of uncertainty” [39] in diagnostic and therapeutic processes by making the meaning of words explicit. It provides a structure that can guide students and physicians in learning to recognize the different ways certainty or uncertainty is implicitly expressed, and how to choose their words carefully. Students, for example, could use this framework to analyze videographed handoffs and study the individual ways of implicit expression of certainty and uncertainty. Such an approach might help them to overcome their fear of seeming uncertain [33] and they could learn how to make uncertainty explicit in their expressions for the sake of patient safety. Currently, communication strategies are mostly rooted in stereotypes transported by the hidden curriculum, such as the misconception that good physicians have to know everything all the time [14]. The new framework could also help teachers in supporting students to express their certainty or uncertainty during the clinical reasoning process more explicitly. Attention should especially be paid to language from the subcategories “questionable”, “incomplete”, “alterable”, and “unreliable”. If implicit uncertainty by attenuation of plain information is not detected by the listener, their clinical reasoning process after a case presentation or handoff, can be faulty and potentially lead to medical errors.

While training programs already exist that focus on interventions reflecting the implications of uncertainty on learners’ emotions, reasoning, decision-making and attitudes [40] there are no reports of training programs with a view on language that explicitly expresses uncertainty. Supporting students on using language that makes their uncertainty explicit might help to address ambiguity in medical education [41]. In a similar way, reflective writing has supported first year medical students to express uncertainty and to emotionally deal with it [42]. Such an approach could be useful to decrease medical students’ and physicians’ intolerance of uncertainty, which has been shown to lead to unnecessary test ordering and reduced compliance with evidence-based guidelines [43]. Teaching the language of uncertainty could also support instruction in clinical reasoning, specifically regarding the differing definitions of truth (coherence and credibility vs. empiric accuracy) which supplement each other in problem solving [44].

Certain limitations and challenges for further research need to be addressed. Our empirically derived framework provides differentiated categories for language, which either attenuate or strengthen plain information and thereby increase uncertainty or certainty. Even though we were able to retrieve a number of samples per category, there might still be further examples, which were not abundant in the material. Our data do not allow any conclusions about the hierarchy of the detected categories and subcategories and support only limited hypotheses about the combination and interaction between language examples from different categories. Even though students from three medical schools with different undergraduate curricula were involved in this study – which we consider a strength – all medical schools were from the same country, and worked within the same health care system. Therefore, the categories might not be generalizable to other countries. Further studies are required to enrich the number of language examples per category and the framework can be used as a starting point for specific differentiation as needed. This research has developed a categorization system, but to study the effects of the different attenuators and strengtheners, recipients of a handoff should also be studied to determine their perception of the reporter’s certainty or uncertainty, and how uncertain they were as a result. By explicitly learning those aspects of language which implicitly increase certainty or uncertainty, students will be able to better go beyond the hidden curriculum in learning professionalism and patient safety.

## Conclusions

Our new, grounded theory derived framework of implicit expressions of uncertainty provides categories of language which attenuate or reinforce plain information, thereby shifting its content implicitly more towards certainty or uncertainty. The framework will support users in analyzing such modifications in communication and in making uncertainty explicit for the sake of patient safety. Medical students and physicians could use it as a learning tool to become aware of their implicit uncertainty and to improve their case presentations and case discussions. The framework provides a basis for further differentiation by identification of implicit expression of uncertainty in different cultural or subject-specific contexts.

## Data Availability

All data and materials are available in the manuscript and can be obtained from the corresponding author upon request.
